# Effects of glia metabolism inhibition on nociceptive behavioral testing in rats

**DOI:** 10.1016/j.dib.2016.02.043

**Published:** 2016-02-26

**Authors:** Yan Lefèvre, Aurélie Amadio, Peggy Vincent, Amélie Descheemaeker, Stéphane H.R. Oliet, Radhouane Dallel, Daniel L. Voisin

**Affiliations:** a Neurocentre Magendie, Inserm U1215, F-33000 Bordeaux, France; b Univ. Bordeaux, F-33000 Bordeaux, France; c Clermont Université, Université d׳Auvergne, NEURO-DOL, BP 10448, F-63000 Clermont-Ferrand, France; d Inserm, U1107, F-63001 Clermont-Ferrand, France

**Keywords:** Fluoroacetate, Intrathecal, Nociception, Rat

## Abstract

Fluoroacetate has been widely used to inhibit glia metabolism *in vivo*. It has yet to be shown what the effects of chronic intrathecal infusion of fluoroacetate on nociceptive behavioral testing are. The effects of chronic infusion of fluoroacetate (5 nmoles/h) for 2 weeks were examined in normal rats. Chronic intrathecal fluoroacetate did not alter mechanical threshold (von Frey filaments), responses to supra-threshold mechanical stimuli (von Frey filaments), responses to hot (hot plate) or cool (acetone test) stimuli and did not affect motor performance of the animals, which was tested with rotarod. This suggests that fluoroacetate at appropriate dose did not suppress neuronal activity in the spinal cord.

**Specifications table**

**Table:** 

Subject area	*Biology*
More specific subject area	*Pain*, *Neuroscience*
Type of data	*Figure*
How data was acquired	*Behavioral testing*
Data format	*Analyzed*
Experimental factors	*Adult rats were* intrathecally infused with fluoroacetate for 2 weeks
Experimental features	Behavioral testing of nociceptive stimuli
Data source location	*Bordeaux*, *France*
Data accessibility	*Data is supplied in the article*

**Value of the data**

• The effects of chronic intrathecal infusion of fluoroacetate on nociceptive behavioral testing were tested in adult rats.
• Intrathecal fluoroacetate administered for 2 weeks in rats did not alter responses to mechanical, hot or cool stimuli and did not affect motor performance assessed with rotarod.
• Chronic intrathecal fluoroacetate at appropriate dose did not impair nociceptive behavioral responses in the normal rat.

## 1 Data

**Fig. 1:**
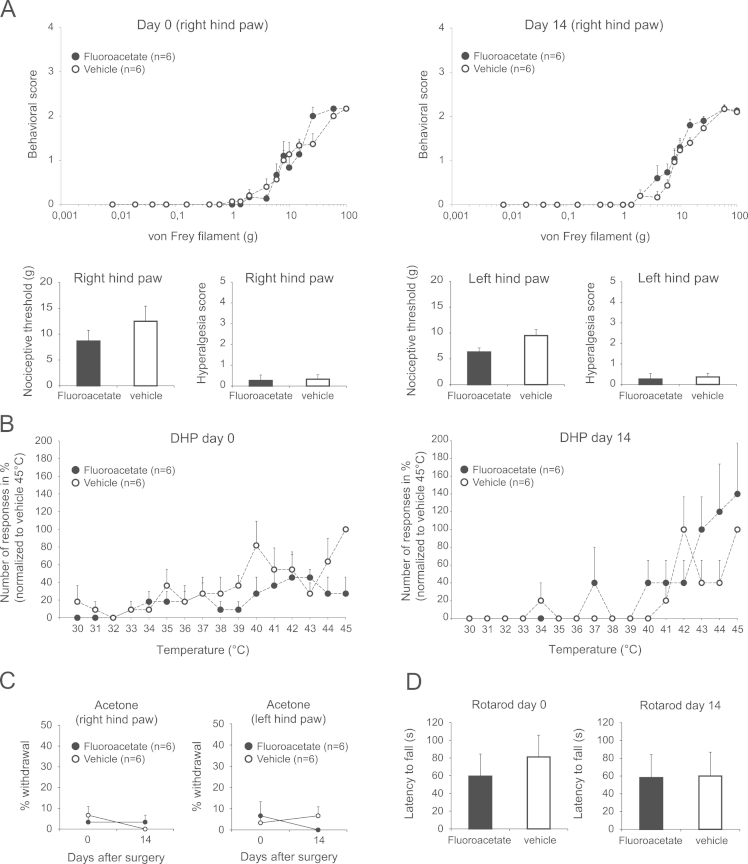
Effect of chronic intrathecal fluoroacetate on responses to mechanical, hot or cool stimuli and motor performance in rats. A. Profiles show mean (±SEM) behavioral scores of rats to von Frey hair stimuli applied on the plantar surface of right hind paws, at day 0 and at day 14 post-infusion of fluoroacetate or vehicle. Bar histograms show mean (±SEM) nociceptive thresholds and hyperalgesia scores for both paws in animals that received fluoroacetate or vehicle. B. Sum of responses (paw licking and withdrawal) for the dynamic hot place (DHP) tests in fluoroacetate treated (filled circles) and vehicle treated (open circles) rats at day 0 (left) and day 14 (right). Responses were normalized to the responses obtained at 45 °C in vehicle treated rats. C. Percentage of ipsilateral foot withdrawal in response to 5 cooling stimuli (drop of acetone) applied on the right or left hind paw at day 0 and at day 14 post-infusion of fluoroacetate or vehicle. D. Motor effects of fluoroacetate as assessed by the rotarod. Bar histograms show mean (±SEM) of the latency to fall in in fluoroacetate treated (filled bars) and vehicle treated (open bars) rats at day 0 (left) and day 14 (right).

We investigated the effects of chronic intrathecal infusion of fluoroacetate on nociceptive behavioral testing in adult rats. Fluoroacetate did not change the profiles of responses to von Frey hair stimuli applied on hind paws (Fig. 1A), did not alter responses to the Dynamic Hot Plate test (Fig. 1B) and to the acetone test (Fig. 1C) and did not affect motor performance of the animals, which was tested with rotarod (Fig. 1D).

## 2 Experimental design, materials and methods

### 2.1 Animals

Adult rats (150–175 g) were obtained from Janvier (France) and maintained in a controlled environment (lights on 07:00–19:00, 22 °C) with food and water freely available. They were housed 2 per cage. All efforts were made to minimize the number of animals used. The experiments followed the ethical guidelines of the International Association for the Study of Pain and the European Community Council directive of 22 September 2010 (2010/63/EU). The project was approved by Bordeaux Ethical Committee (CEEA50) under No. 50120169-A. Rats were acclimatized for 4 days to the animal facility and for 5 days to manipulations and devices prior to behavioral studies.

### 2.2 Surgery and drugs

Fluoroacetate or vehicle (artificial cerebrospinal fluid, aCSF) was administered through intrathecal catheters that were surgically implanted above the lumbar part of the spinal cord in animals at day 0 under isoflurane anesthesia. Catheters were connected to an Alzet osmotic pump delivering 0.5 μL/h. The procedure is described in [1]. Fluoroacetate (5 nmoles/h, Sigma, Illkirch, France) was dissolved in aCSF and administered chronically for 14 days [1]. In control experiments, only vehicle was injected.

### 2.3 Behavioral studies

Mechanical allodynia and hyperalgesia were measured at the hind paw using score values calculated from responses to von Frey filament stimulation according to the method adapted from Ducourneau et al. [2] and described in [1].

The first mean score value above detection was 1.2 and was used to obtain the nociceptive response-threshold. Accordingly, this score value corresponds to the stimulus intensity at which more than 50% of withdrawal responses could be observed over 5 responses. The first von Frey hair value giving a mean score above 1.2 was considered the nociceptive response-threshold.

To quantify mechanical hyperalgesia, we used a hyperalgesia score calculated as the sum of the differences between values obtained for each von Frey hair after and before (day 0) surgery and taken from above the value of the initial nociceptive threshold [1].

To quantify mechanical allodynia, shifts in nociceptive response-threshold were measured.

Heat allodynia and hyperalgesia were assessed by using a computer-controlled hot plate analgesia meter (Bioseb, France). Animals were placed on the dynamic hot plate at 30 °C and the plate temperature increased up to 45 °C with 1 °C min^−1^ increment. During each degree interval, we scored hind paw lickings and paw withdrawals. The sum of the paw lickings and paw withdrawals was used as index of nociceptive response [3]. For each recording day, nociceptive responses were expressed as percentage of the maximal nociceptive responses obtained at 45 °C in control rats.

Cold allodynia was assessed by application of a drop of acetone via a syringe to the back of the plantar surface of the hind paw, alternating between ipsilateral and contralateral sides. A total of five applications were made to each hind paw with at least 5 min separating each. The number of withdrawals was expressed as a percentage of maximum response.

To investigate the motor effects of fluoroacetate, rats were tested on the accelerating rotarod. All values are expressed as mean±SEM. To assess changes after drug administration unpaired or paired (as appropriate) *t*-tests were performed. Differences were considered significant at *p*<0.05. In the acetone test, responses of both hind paws were pooled together for statistical comparison.
